# Antitumor activity of the multikinase inhibitor regorafenib in patient-derived xenograft models of gastric cancer

**DOI:** 10.1186/s13046-015-0243-5

**Published:** 2015-10-29

**Authors:** Hung Huynh, Richard Ong, Dieter Zopf

**Affiliations:** Humphrey Oei Institute of Cancer Research, National Cancer Centre, 11 Hospital Drive, Singapore, 169610 Singapore; Bayer Pharma AG, Müllerstraße 178, 13353 Berlin, Germany

**Keywords:** Regorafenib, Gastric cancer, Antitumor, Angiogenesis, Patient-derived xenograft models

## Abstract

**Background:**

Unresectable gastric cancer is associated with poor outcomes, with few treatment options available after failure of cytotoxic chemotherapy. Clinical trials of targeted therapies have generally shown no survival benefit in gastric cancer, with the exceptions of the antibodies ramucirumab (anti-VEGFR2) and trastuzumab (anti-HER2/neu). Given the efficacy of the multikinase inhibitor regorafenib in other gastrointestinal tumors, we investigated its potential in gastric cancer.

**Methods:**

The antitumor activity of oral regorafenib was assessed in eight murine patient-derived gastric cancer xenograft models. Dose–response experiments assessed the efficacy and tolerability of oral regorafenib 5, 10, and 15 mg/kg/day in two models, with 10 mg/kg/day selected for further investigation in all eight models. Tumor weight and volume was monitored during treatment; tumor cell proliferation, angiogenesis, apoptosis, and intracellular signaling were assessed using immunohistochemistry and Western blotting of total tumor lysates at the end of treatment.

**Results:**

Regorafenib showed dose-dependent inhibition of tumor growth and was well tolerated, with no significant decreases in bodyweight or evident toxicity. Regorafenib 10 mg/kg/day significantly inhibited tumor growth in all eight models (72 to 96 %; all *p* < 0.01), resulting in reduced tumor weight versus vehicle controls. Regorafenib reduced tumor angiogenesis 3- to 11-fold versus controls in all models (all *p* < 0.05), reduced tumor proliferation 2- to 5-fold in six of the eight models (all *p* < 0.05), and induced apoptosis in seven models.

**Conclusion:**

Regorafenib was effective in patient-derived models of gastric cancer of different histological subtypes, with inhibition of tumor growth, angiogenesis, and tumor-cell proliferation observed in almost all models. These findings are consistent with the observed activity of regorafenib in preclinical models of other gastrointestinal tumors, and support further clinical investigation in gastric cancer.

**Electronic supplementary material:**

The online version of this article (doi:10.1186/s13046-015-0243-5) contains supplementary material, which is available to authorized users.

## Introduction

Gastric cancer is the fifth most common malignancy worldwide, with an estimated 950,000 new cases in 2012; approximately two thirds of cases occur in men [1, 2]. Mortality statistics are even more striking, with more than 720,000 deaths due to gastric cancer estimated to occur each year, making it the third most common cause of cancer-related death [1, 2]. Only 30 % of cases occur in developed countries, while the highest incidence is in Eastern Asia, particularly China, accounting for 50 % of patients [1, 2]. A number of risk factors for the development of gastric cancer have been identified, the most important of which is infection with *Helicobacter pylori* [3].

Surgical resection is the first choice of treatment for early-stage gastric cancer [4]; however, many cases are locally advanced or metastatic at the time of diagnosis and are thus unresectable [5]. Although a number of cytotoxic agents have been found effective in this indication, treatment options for patients whose disease progresses on chemotherapy are limited [4], resulting in an overall 5-year survival rate of just 28 % [5].

The presence of gain-of-function mutations affecting receptor tyrosine kinases (RTKs) is associated with poor prognosis in patients with gastric cancer [6, 7]. In an effort to provide additional treatment options in this patient group, a variety of targeted therapies have been investigated. Potential molecular targets identified include RTKs involved in angiogenesis and tumor proliferation, such as vascular endothelial growth factor (VEGF), angiopoietin, platelet-derived growth factor (PDGF), fibroblast growth factor (FGF) receptors, and HER2/neu [6, 8–10]. Interestingly, some of the receptors identified as potential targets have overlapping intracellular signal transduction cascades, notably the PI3K/AKT/mTOR and MAPK pathways [11–14]. Activation of these signaling cascades is associated with increased tumor-cell proliferation and survival, as well as inhibition of apoptosis [15, 16].

Clinical trials of targeted therapies in gastric cancer have met with varying levels of success. Studies of bevacizumab, cetuximab, panitumumab, and everolimus have failed to show a significant survival benefit versus varying control treatments, and a phase II trial of sunitinib failed to meet its primary endpoint [17–22]. However, the anti-VEGF receptor 2 (VEGFR2) antibody ramucirumab improved survival compared with placebo in a phase III trial [23] and has been approved for advanced gastric cancer by the US Food and Drug Administration and the European Medicines Agency. Addition of the anti-HER2/neu monoclonal antibody trastuzumab to chemotherapy has also been shown to provide benefit versus chemotherapy alone in patients with HER2/neu-positive tumors [24].

Regorafenib is a multikinase inhibitor with activity at a range of protein kinases involved in oncogenesis (KIT, RET, and RAF), angiogenesis (VEGFR1–3 and TIE2), and maintenance of the tumor microenvironment (PDGFR and FGFR) [25]. Regorafenib has demonstrated efficacy in phase III trials in patients with metastatic colorectal cancer (CRC) [26, 27] and advanced gastrointestinal stromal tumors (GIST) [28] and has been approved in these indications in a number of countries. Given the wide range of kinases inhibited by regorafenib and its clinical efficacy in other gastrointestinal tumors, we investigated its antitumor activity in patient-derived xenograft (PDX) models of gastric cancer.

## Methods

### Reagents

Antibodies against Bim, cleaved poly(ADP ribose) polymerase (PARP), AKT, p-Ser^473^ AKT, p-Thr^202^/Tyr^204^ ERK1/2, p-Ser^10^ histone H3, S6R, p-Ser^235/236^ S6R, Rb, p-Ser^780^ Rb, p-Ser^807/811^ Rb, VEGFR2, p-Tyr^951^ VEGFR2, p90RSK1–3, p-Thr^359^/Ser^363^ p90RSK, p70S6K, p-Thr^421^/Ser^424^ p70S6K, p-Tyr^15^ CDC-2, p-Thr^14^/Tyr^15^ CDK-2, 4EBP1, p-Thr^70^ 4EBP1, and TIE2 were obtained from Cell Signaling Technology. Antibodies against BAD, p21, CD-31, CDK-2, CDK-4, CDC-2, cyclin B1, ERK1/2, p27, survivin, and α-tubulin were obtained from Santa Cruz. Triton X100, NaCl, and NP-40 were obtained from Merck KGaA. EDTA, sodium orthovanadate, and Tris-base were from Sigma-Aldrich. Tween-20 was purchased from Promega Corporation.

Regorafenib was dissolved in dimethyl sulfoxide to create a stock solution with a concentration of 100 mg/mL. To achieve the solution with the final concentration for administration, 0.1 mL of the regorafenib stock solution (or dimethyl sulfoxide for the control group) was further diluted in vehicle (4 mL of polyethylene glycol 300 and 3.9 mL of 30 % Captisol [purchased from CyDex] in water).

### Patient-derived xenografts

Animal experiments were approved by the ethics board at the National Cancer Centre of Singapore and Singapore General Hospital. All mice were maintained according to the Guide for Care and Use of Laboratory Animals, published by the US National Institutes of Health [29]. Animals were provided with sterilized food and water *ad libitum*, and were housed in negative-pressure isolators with 12-h light/dark cycles.

Xenograft experiments were performed with male severe combined immunodeficiency (SCID) mice (Animal Resources Centre). Eight patient-derived gastric cancer PDX models (GC09-0109, GC28-1107, GC22-0808, GC30-0309, GC10-0608, GC17-0409, GC05-0208B, and GC23-0909) were used to establish subcutaneous tumors in mice aged 9–10 weeks. Tumor model histology and mutation status are shown in Additional file 1: Table S1.

### Antitumor activity *in vivo*

For dose response and tolerability analyses, mice bearing GC09-0109 and GC28-1107 tumor xenografts were given oral vehicle or regorafenib 5, 10, or 15 mg/kg/day. Each treatment group comprised 15 or 16 mice. Treatment was started when tumors reached approximately 150 to 200 mm^3^. Tumors were measured bidimensionally and their volume was calculated using the formula: (length) × (width^2^) × (π/6). Mice were killed at the end of the study; tumor weight and bodyweight were recorded, and tumors were preserved for further analysis.

For the assessment of antitumor activity in additional tumor models, xenografts were grown subcutaneously in mice (14 to 20 mice per group) to a size of approximately 200 to 300 mm^3^. Mice were then given daily oral doses of either 200 μL of vehicle or regorafenib 10 mg/kg, with the last dose given 2 h before death. Tumor volumes were determined as in the dose–response experiments. A portion of each tumor was fixed in paraformaldehyde and embedded in paraffin, with further portions snap frozen for tumor lysate generation and cryopreserved for immunohistochemistry (IHC).

### Western blot analysis

To investigate changes in levels of phosphorylated and total proteins identified as targets of regorafenib or with roles in tumor cell proliferation, apoptosis, cell cycle regulation, and survival, three to four randomly selected independent tumors from vehicle and drug-treated mice were combined and homogenized in lysis buffer (0.5 % Triton X100; 150 mMol/L NaCl; 10 mMol/L EDTA; 2 mMol/L sodium orthovanadate; 0.5 % NP-40). Protein concentration was determined by Bio-Rad protein assay (Bio-Rad Laboratories). Eighty micrograms of total lysate per tumor sample preparation were analyzed by Western blot. Blots were incubated with primary antibodies diluted in TBST (20 mMol/L Tris, pH 7.6, 150 mMol/L NaCl and 0.1 % Tween-20) containing 1 % nonfat dry milk and a 1:7500 dilution of horseradish peroxidase-conjugated secondary antibodies. All primary antibodies were then visualized with a chemiluminescent detection system (Amersham, Pharmacia Biotech).

### Immunohistochemistry and histological staining

Fifteen micron sections of optimal cutting temperature compound-embedded (Tissue-Tek; Sakura Finetek) tumors were immunostained with anti-CD31 antibodies to assess microvessel density (MVD). To quantify MVD, the number of immunostained vessels in ten 0.159 mm^2^ fields at a magnification of × 100 from ten randomly selected tumors in each group was counted. Five micrometer sections of paraffin-embedded tumor tissue were immunostained with anti-p-Ser^10^ histone H3 or cleaved PARP antibodies to assess tumor-cell proliferation and apoptosis, respectively, based on the percentage of p-Ser^10^ histone H3-positive and cleaved PARP-positive cells per ≥500 cell region, respectively. Three tumors per treatment and four regions per tumor were analyzed for tumor cell proliferation and apoptosis. Induction of apoptosis was defined as a two-fold or greater increase in the proportion of cells identified as cleaved PARP-positive in tumors from regorafenib-treated mice compared with tumors from vehicle-treated animals. Images were recorded using an Olympus BX60 microscope equipped with an Olympus DP11 camera. All experiments were performed in triplicate.

Tumor necrosis was assessed by microscopic examination of hematoxylin and eosin (H&E)-stained tumor sections, with ten random fields examined at a × 100 magnification. Tumor necrosis was only qualitatively assessed.

### Statistical analysis

Differences in tumor weight at death, p-Ser^10^ histone H3 index, mean MVD, and cleaved PARP-positive cells were compared by analysis of variance or Student’s *t-*test. Significance was established at *p <* 0.05 for all statistical analyses.

## Results

### Regorafenib inhibits growth of gastric cancer xenografts in a dose-dependent manner

**Fig. 1:**
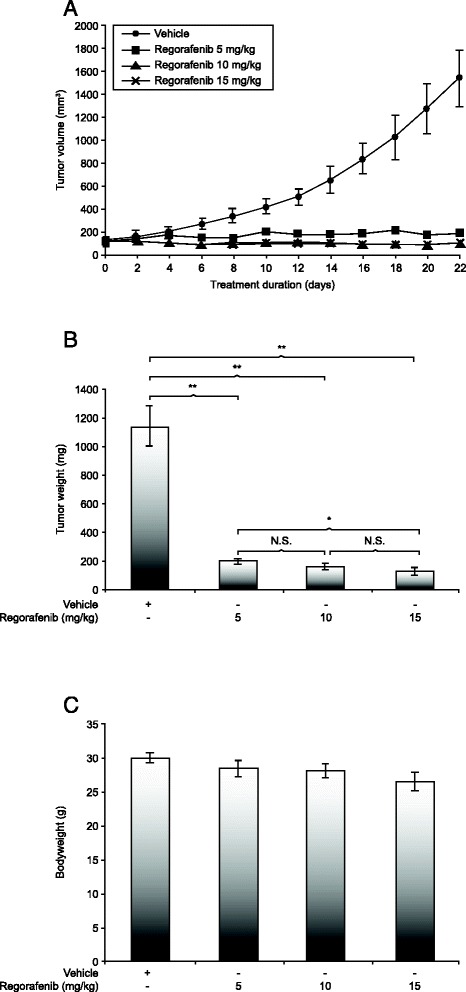
Regorafenib dose-dependently inhibits growth of patient-derived xenografts. The effects of regorafenib 5, 10, and 15 mg/kg/day on tumor growth inhibition (**a**), tumor weight (**b**), and bodyweight (**c**) in xenograft model GC09-0109 are shown. Data are mean ± standard error (*, *p* < 0.01; **, *p* < 0.001; N.S., not significant)

Regorafenib 5, 10, or 15 mg/kg/day was administered for 22 days to mice with GC09-0109 and GC28-1107 xenografts, with tumor growth inhibition assessed by comparison of the tumor weights of vehicle- and regorafenib-treated animals. In the GC09-0109 model, regorafenib was associated with 81 to 88 % inhibition of tumor growth compared with vehicle-treated animals (*n* = 15 per group; Fig. 1a), while the GC28-1107 model showed 72 to 88 % inhibition of tumor growth compared with vehicle-treated controls (*n* = 16 per group; Additional file 2: Figure S1a). Tumors from regorafenib-treated mice weighed dose-proportionately less than those from vehicle-treated mice (all *p <* 0.05; Fig. 1b and Additional file 2: Figure S1b). No significant loss of bodyweight (Fig. 1c and Additional file 2: Figure S1c) or signs of toxicity were observed in any of the treatment groups.

On the basis of the observed tumor growth inhibition and tolerability profile of regorafenib in the GC28-1107 xenograft line, and consistent with previous studies [25, 30], the 10 mg/kg/day dose was selected for further investigation in other gastric cancer xenograft lines.

### Regorafenib 10 mg/kg/day inhibits tumor growth in gastric cancer xenograft models

**Fig. 2:**
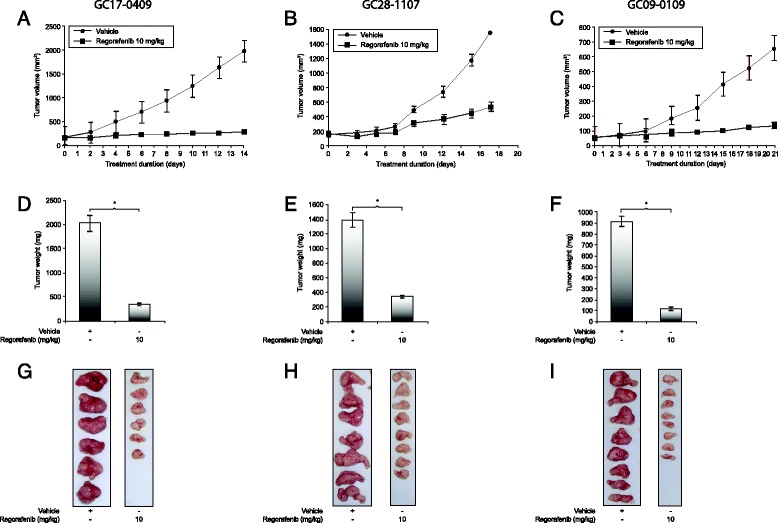
Regorafenib 10 mg/kg/day significantly inhibits growth of patient-derived xenografts. The effects of regorafenib 10 mg/kg/day on tumor growth inhibition (**a**, **b**, and **c**), tumor weight (**d**, **e**, and **f**), and representative tumors (**g**, **h**, and **i**) are demonstrated in xenograft models GC17-0409, GC28-1107, and GC09-0109. Data are mean ± standard error (*, *p* < 0.001)

Regorafenib 10 mg/kg/day inhibited tumor growth compared with vehicle in all gastric cancer xenograft models, with reductions in tumor weight of 72 to 96 % (all *p <* 0.01; Fig. 2, Additional file 3: Figure S2, and Table 1). The potent antitumor activity appeared to be independent of the histological subtypes of the tumor models, which encompass tumors of intestinal, diffuse, mixed, and tubular origin (Additional file 1: Table S1). Although the study had a small sample size, there appeared to be no correlation between mutational status, including KRAS and PDGFR-α mutation status, and tumor growth inhibition (Additional file 1: Table S1). As in the dose–response experiments, no significant loss of bodyweight or signs of toxicity were observed in any of the treatment groups (data not shown).

**Table 1:** Regorafenib inhibits the growth of patient-derived gastric cancer xenograft models

Xenograft model	Treatment	Mean ± SE tumor weight (g)	Tumor growth inhibition (%)^a^	*p* value versus vehicle
GC09-0109	Vehicle	1.14 (0.12)		
	Reg 5	0.21 (0.03)	81.4	<0.001
	Reg 10	0.17 (0.02)	84.9	<0.001
	Reg 15	0.14 (0.02)	88.1	<0.001
GC28-1107	Vehicle	1.72 (0.16)		
	Reg 5	0.48 (0.06)	72.4	<0.001
	Reg 10	0.39 (0.06)	77.6	<0.001
	Reg 15	0.21 (0.03)	88.0	<0.001
GC09-0109	Vehicle	0.92 (0.05)		
	Reg 10	0.14 (0.01)	84.9	<0.01
GC22-0808	Vehicle	2.55 (0.27)		
	Reg 10	0.11 (0.01)	95.9	<0.001
GC17-0409	Vehicle	2.03 (0.22)		
	Reg 10	0.36 (0.05)	82.4	<0.001
GC10-0608	Vehicle	3.11 (0.35)		
	Reg 10	0.31 (0.04)	90.0	<0.001
GC28-1107	Vehicle	1.40 (0.12)		
	Reg 10	0.40 (0.02)	71.5	<0.001
GC23-0909	Vehicle	1.73 (0.11)		
	Reg 10	0.22 (0.02)	87.0	<0.001
GC30-0309	Vehicle	3.48 (0.28)		
	Reg 10	0.59 (0.07)	83.0	<0.001
GC05-0208B	Vehicle	2.17 (0.10)		
	Reg 10	0.49 (0.03)	77.6	<0.001

Reg 5: regorafenib 5 mg/kg/day; Reg 10: regorafenib 10 mg/kg/day; Reg 15: regorafenib 15 mg/kg/day

^a^Tumor growth inhibition based on the difference in tumor weight between vehicle and regorafenib

### Regorafenib reduces tumor angiogenesis

**Fig. 3:**
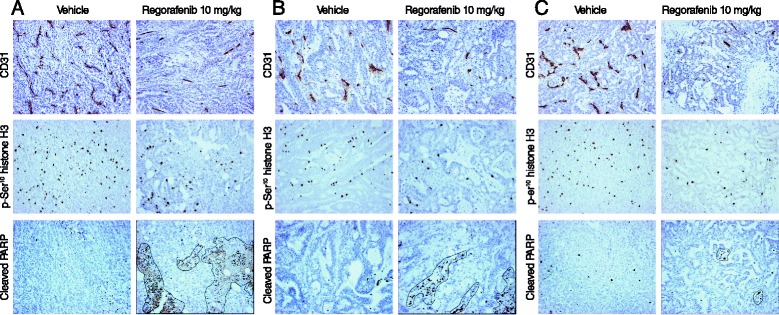
Regorafenib 10 mg/kg/day inhibits tumor angiogenesis and tumor cell proliferation, and induces apoptosis. The effects of regorafenib on tumor angiogenesis (CD31 expression), cell proliferation (p-Ser^10^ histone H3 expression), and apoptosis (cleaved PARP expression) are demonstrated in xenografts GC17-0409 (diffuse histology; **a**), GC28-1107 (more intestinal; **b**), and GC09-0109 (tubular histology; **c**). Dotted lines denote areas of necrosis. PARP: poly(ADP ribose) polymerase

Regorafenib 10 mg/kg/day significantly reduced MVD in all gastric cancer xenograft models compared with vehicle (all *p <* 0.05), as assessed by binding of anti-CD31 antibodies. Mean MVDs in regorafenib-treated mice were three- to eleven-fold lower than in vehicle-treated mice (Fig. 3 and Table 2). Tumors from regorafenib-treated mice generally had a pale appearance, consistent with reduced MVD and poor vascularization (Fig. 2g–i). IHC of the GC05-0208B model showed that TIE2 was expressed in host-derived stromal cells and blood vessels, but not in tumor cells. TIE2 immunostaining did not appear to differ between regorafenib- and vehicle-treated tumors (Additional file 4: Figure S3).

**Table 2:** Regorafenib 10 mg/kg/day reduces mean microvessel density, cell proliferation (p histone H3 Ser^10^-positive cells) and apoptosis (cleaved PARP-positive cells) in patient-derived gastric cancer xenograft models

	Mean (SE) microvessel density^a^			Mean (SE) p-histone H3 Ser^10^-positive cells, %			Mean (SE) cleaved PARP-positive cells, %		
Xenograft model	Vehicle	Reg 10	Fold difference	Vehicle	Reg 10	Fold difference	Vehicle	Reg 10	Fold difference
GC09-0109	20.0 (6.0)	2.0 (0.5)*	10.0	8.3 (1.6)	2.1 (0.7)*	4.0	1.2 (0.5)	2.1 (0.6)	1.8
GC28-1107	11.4 (1.8)	2.4 (0.8)*	4.8	4.6 (0.4)	1.4 (0.6)*	3.3	0.3 (0.1)	7.3 (1.5)*	24.3
GC22-0808	23.0 (7.0)	2.1 (0.7)*	11.0	8.9 (2.1)	8.2 (1.4)	1.1	2.7 (1.5)	6.8 (2.4)*	2.5
GC30-0309	16.0 (4.0)	5.0 (2.0)*	3.2	17.8 (8.0)	5.3 (2.0)*	3.4	1.4 (0.7)	17.2 (4.0)*	12.3
GC10-0608	24.0 (5.0)	2.7 (1.0)*	8.9	10.3 (3.0)	2.1 (0.4)*	4.9	0.9 (0.4)	4.8 (1.4)*	5.3
GC17-0409	28.0 (7.0)	5.0 (1.4)*	5.6	15.3 (5.0)	4.8 (1.1)*	3.2	0.4 (0.2)	31.0 (10.0)*	77.5
GC05-0208B	43.0 (11.0)	4.0 (1.2)*	10.8	4.8 (0.9)	3.1 (0.8)	1.5	3.1 (0.9)	9.9 (3.0)*	3.2
GC23-0909	16.1 (2.8)	5.2 (1.0)*	3.1	13.9 (2.8)	5.7 (1.7)*	2.4	0.3 (0.1)	7.3 (1.9)*	24.3

PARP: poly-(ADP ribose) polymerase; Reg 10: regorafenib 10 mg/kg/day; SE: standard error

*p < 0.05 versus vehicle

^a^Number of immunostained vessels in ten 0.159 mm^2^ fields from ten randomly selected tumors at a magnification of × 100

**Fig. 4:**
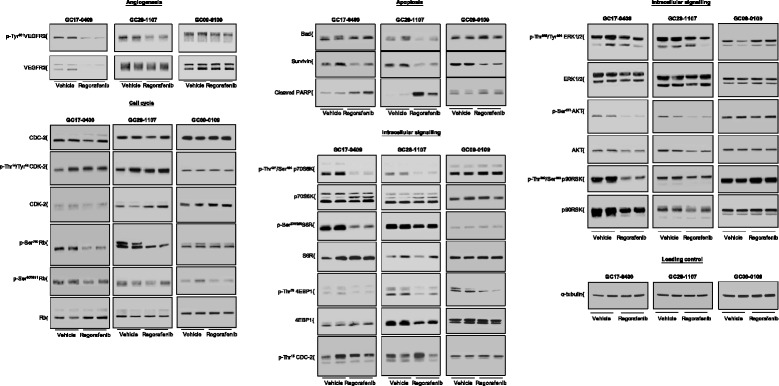
Effects of regorafenib on protein expression and phosphorylation in GC17-0409, GC28-1107, and GC09-0109 xenografts. Western blots were performed using two pooled lysates of three to four individual tumors each per group

Analysis of changes in VEGFR2 expression in pooled tumor lysates produced heterogeneous results, with reduced total or p-Tyr^951^ VEGFR2 levels in GC17-0409 and GC28-1107 tumors, respectively, and no apparent changes in p-Tyr^951^ VEGFR2 levels in GC09-0109 tumors (Fig. 4). These disparate results may be related to histological or spatio-temporal differences between VEGFR2 inhibition and reductions in MVD.

### Regorafenib inhibits tumor cell proliferation

Tumor cell proliferation, based on the proportion of p-Ser^10^ histone H3-positive cells, was two- to five-fold lower in six xenograft models after treatment with regorafenib 10 mg/kg/day than in tumors from vehicle-treated mice (*p <* 0.05; Fig. 3 and Table 2).

Pooled tumor lysates from regorafenib- and vehicle-treated mice were analyzed for the effects of regorafenib on selected proteins involved in the MAPK and AKT/mTOR signaling pathways and for selected proteins involved in the cell cycle (Fig. 4). No overt effects were observed on p-ERK levels in the GC17-0409, GC28-1107, and GC09-0109 models. Analysis of p-AKT and some of its downstream target proteins produced variable results. Whereas no changes were observed in the GC09-0109 model, levels of p-Ser^473^ AKT, p-Thr^359^/Ser^363^ p90RSK, p-Thr^421^/Ser^424^ p70S6K, p-Ser^235/236^ S6R, and p-Thr^70^ 4EBP1 appeared to be consistently reduced in the GC17-0409 and GC28-1107 models, suggesting that the AKT/mTOR pathway plays a role in the growth-inhibiting effects of regorafenib in these models. Moderate or no effects were observed on levels of cell cycle proteins such as p-Tyr^15^ CDC-2, p-Thr^14^/p-Tyr^15^ CDK-2, p-Ser^780^ RB, and p-Ser^807/811^ RB, indicating that these mechanisms do not play a role in the antiproliferative effect of regorafenib.

### Regorafenib induces apoptosis and central necrosis

Regorafenib 10 mg/kg/day induced apoptosis in seven of the eight gastric cancer xenograft models (all *p <* 0.05 versus tumors from vehicle-treated animals; Table 2 and Fig. 3) measured by detection of cells staining positive for cleaved PARP on IHC. Differences in the number of cleaved PARP-positive cells in tumors from regorafenib- versus vehicle-treated animals ranged from approximately two-fold to more than 75-fold (Table 2). Western blot of pooled tumor lysate confirmed that, compared with tumors from vehicle-treated mice, tumors from regorafenib-treated mice consistently showed elevated levels of cleaved PARP in the GC17-0409, GC28-1107, and GC09-0109 models, although the levels of elevation were variable (Fig. 4), coupled with decreased levels of the antiapoptotic protein survivin. No obvious changes in levels of the proapoptotic protein BAD were detected.

In addition to induction of apoptosis, central necrosis was observed in H&E-stained sections of tumors from all eight xenograft models following treatment with regorafenib (Fig. 3 and data not shown). Areas of necrosis were preferentially associated with apoptosis.

## Discussion

Expression of VEGF is strongly correlated with tumor progression and poor prognosis in gastrointestinal malignancies, including gastric cancer [31], with an association between VEGF expression, increased MVD, and decreased survival established in previous studies [10, 32]. Preclinical studies of VEGFR-targeting agents in gastric cancer have shown significant antitumor effects [33, 34], and a clinical trial with the VEGFR2 antibody ramucirumab monotherapy has demonstrated survival benefits over placebo for patients with advanced gastric cancer, validating VEGFR2 as a relevant therapeutic target in gastric cancer [23]. However, overall survival gains after ramucirumab treatment were moderate and the response rate was low [23], which indicates a need for additional antiangiogenic approaches.

This study was performed to assess the antitumor activity of the multikinase inhibitor regorafenib, a known potent inhibitor of VEGFR kinases in gastric cancer xenografts, and to investigate the underlying antitumor mechanisms. Our findings show that all eight patient-derived xenograft models investigated in the current study respond favorably to regorafenib, with tumor growth inhibition of 72 to 96 % at a dose of 10 mg/kg/day in a variety of histological subtypes. At this dose, regorafenib exposure and C_max_ in mice are comparable to those observed in humans after 21 days of treatment with regorafenib 160 mg/day [35], a dose which has demonstrated efficacy in patients with CRC and GIST [26–28].

Analysis of the mechanisms by which regorafenib inhibited tumor growth inhibition showed a pronounced antiangiogenic effect in xenografts from all regorafenib-treated mice, as measured by MVD reduction versus vehicle-treated animals. Tumors from vehicle-treated animals were well vascularized, as judged by both measured MVD and visual appearance (Figs. 2 and 3 and Table 2). Elevated MVD has previously been detected in clinical samples of diffuse- versus intestinal-type tumors [10, 32], which was not apparent in our vehicle-treated xenografts. A stronger antiangiogenic effect with regorafenib treatment was noticed in intestinal than in diffuse-type tumor models, but did not translate into differences in antitumor activity. Consistent with the antiangiogenic effects detected by IHC, levels of phosphorylated or total VEGFR2 protein were reduced in pooled tumor lysates from some models (Fig. 4).

Regorafenib also inhibited cell proliferation, as shown by the significant decrease in the proportion of p-Ser^10^ histone H3-positive cells in all but two models, both of which were of intestinal origin. However, there was no correlation between the antiproliferative and antitumor effects, similar to what was observed with the antiangiogenic effects. Ser^10^ of histone H3 is phosphorylated by mitogen- and stress-activated kinase 1, Aurora B, or checkpoint kinase 1 [36]; none of these kinases is significantly inhibited by regorafenib in biochemical assays [25], precluding a direct antiproliferative activity of regorafenib by inhibition of Ser^10^ histone H3 phosphorylation. In Western blots, no effects were observed on proteins associated with the cell cycle, such as cyclin-dependent kinases 2 (Fig. 4), cyclin-dependent kinases 1, 4, and 6 (data not shown), and the RB protein (Fig. 4). Although not systematically analyzed, regulatory proteins such as cyclin B1 and the cyclin inhibitors p21 and p27 were not affected (data not shown). Given these findings, more detailed research is required to provide a molecular explanation for the antiproliferative effect of regorafenib.

Gastric cancer cell apoptosis was induced by regorafenib through the caspase-mediated mitochondrial pathway, demonstrated by the increased proportion of caspase-cleaved PARP-positive tumor cells and elevated levels of cleaved PARP in tumor lysates (Fig. 4 and Table 2). The extent of apoptosis induction varied widely and was strongest in diffuse- and mixed-type tumor models; however, there was no correlation with tumor growth inhibition. Small reductions in levels of the antiapoptotic protein survivin were consistently observed in all models investigated (Fig. 4 and data not shown), but levels of the proapoptotic protein BAD were not affected. High survivin expression has been correlated with poor prognosis in gastric cancer [37], suggesting that regorafenib-induced reductions could contribute to the antitumor activity of regorafenib. Expression of another proapoptotic protein, PUMA (p53 upregulated modulator of apoptosis), has also been found to be downregulated in gastric cancer [38]. PUMA expression was recently shown to be upregulated by regorafenib in CRC cells [39], raising the possibility that regorafenib may have a similar effect in gastric cancer. In light of the multiple pathways that appear to play a role in apoptosis, it may be necessary to take an integrated systems approach covering the entire apoptosis network, as was used by Lindner et al*.* [40], to better understand the role of apoptosis in the antitumor activity of regorafenib.

The effects of regorafenib on gastric cancer xenografts in the current study are consistent with the findings of previous preclinical studies of regorafenib, including xenograft studies in other gastrointestinal tumor types. At doses of 10 to 30 mg/kg/day, regorafenib inhibited tumor growth by up to 75 % versus vehicle in various CRC tumor models, including subcutaneous xenografts of the tumor cell line Colo-205, five of seven CRC PDX models, and an orthotopic CRC model derived from the murine cell line CT26 [25, 30, 41]. Tumor regression was also observed in a GIST PDX model [42]. Analysis of angiogenesis in the CT26, Colo-205, and Co5896 CRC PDX models and the GIST PDX model showed a significant reduction in tumor vessel area or vessel number in regorafenib-treated xenografts, assessed by CD31 staining, while no significant change in microvessel area was observed in the regorafenib-refractory Co8541 CRC model [25, 30, 41, 42]. These previous results, in addition to those from the current study, suggest that antiangiogenesis is one of the main drivers of the antitumor activity of regorafenib in gastrointestinal tumors such as CRC, GIST, and gastric cancer, with further support from the observation of central necrosis in all of the gastric cancer models of this study (Fig. 3 and data not shown) and in a GIST PDX model [42]. Although not specifically investigated here, induction of hypoxia could lead to apoptosis, which would also explain the apoptotic events observed in this study. Regorafenib has been shown to induce apoptosis in a murine CT26 CRC model, with an approximately 18-fold increase in apoptosis observed in regorafenib-treated mice compared with controls [41]; however, no effects were observed in the GIST PDX model [42].

## Conclusion

In summary, regorafenib appears to be effective in PDX models of gastric cancer, resulting in significant inhibition of tumor growth, angiogenesis, and tumor-cell proliferation, as well as induction of apoptosis. Given these findings, regorafenib warrants further investigation in this indication in clinical studies. Indeed, regorafenib was active in a recent randomized, double-blind, placebo-controlled phase II trial in patients with refractory advanced esophago-gastric cancer (INTEGRATE) [43], with significantly longer progression-free survival observed in the regorafenib group versus placebo.

## Additional files

Additional file 1: Table S1. Tumor xenograft model histology and mutation status. (PDF 11 kb) Additional file 2: Figure S1. Dose-dependent effects of regorafenib 5, 10, and 15 mg/kg/day on tumor growth inhibition (A), tumor weight (B), and bodyweight (C) in xenograft model GC28-1107. (PDF 143 kb) Additional file 3: Figure S2. Effects of regorafenib 10 mg/kg/day on tumor growth in xenografts GC22-0808, GC23-0909, GC30-0309, GC-10-0608, and GC05-0208B. (PDF 75 kb) Additional file 4: Figure S3. Effects of regorafenib on TIE2 expression in gastric cancer xenograft model GC05-0208B. (PDF 94 kb)
