# Supplementary material for: Antitumor activity of the multikinase inhibitor regorafenib in patient-derived xenograft models of gastric cancer
Source: J Exp Clin Cancer Res. 2015 Oct 29;34:132. doi: 10.1186/s13046-015-0243-5 (PMC4625870; doi:10.1186/s13046-015-0243-5)
Supplement: Additional file 1: Table S1. — Tumor xenograft model histology and mutation status. (PDF 11 kb) [file 13046_2015_243_MOESM1_ESM.pdf]

Huynh H, Ong R, Zopf D. Antitumor Activity of the Multikinase Inhibitor Regorafenib in Patient-Derived Xenograft Models of Gastric Cancer

Supplementary Table 1. Tumor xenograft model histology and mutation status

**Xenograft model**	**Histological features**	**Mutation status**
GC09-0109	Tubular	*PDGFRA-D842_S847>EA*
GC28-1107	More intestinal than diffuse	*FBX4-S8R; PIK3CA-H1047R*
GC22-0808	Intestinal	*PIK3CA-E545G; KRAS-G13D*
GC30-0309	Diffuse	*FBX4-G30N; TP53-R273C*
GC10-0608	Mixed diffuse and intestinal	*TP53-R273C; TP53-R306*
GC17-0409	Diffuse	*PDGFRA-D842_S847>EA*
GC05-0208B	Intestinal	*KRAS-G12A*
GC23-0909	Diffuse	*BRAF-R444Q; KRAS-G12D; PIK3CA-C420R*
