# Supplementary material for: Antitumor activity of the multikinase inhibitor regorafenib in patient-derived xenograft models of gastric cancer
Source: J Exp Clin Cancer Res. 2015 Oct 29;34:132. doi: 10.1186/s13046-015-0243-5 (PMC4625870; doi:10.1186/s13046-015-0243-5)

Huynh H, Ong R, Zopf D. Antitumor Activity of the Multikinase Inhibitor Regorafenib in Patient-Derived Xenograft Models of Gastric Cancer

Supplementary Figure 1. Dose-dependent effects of regorafenib 5, 10, and 15 mg/kg/day on tumor growth inhibition (A), tumor weight (B), and bodyweight (C) in xenograft model GC28-1107 (*, $P < 0.001$; N.S., not significant). Data shown are mean ± standard error.

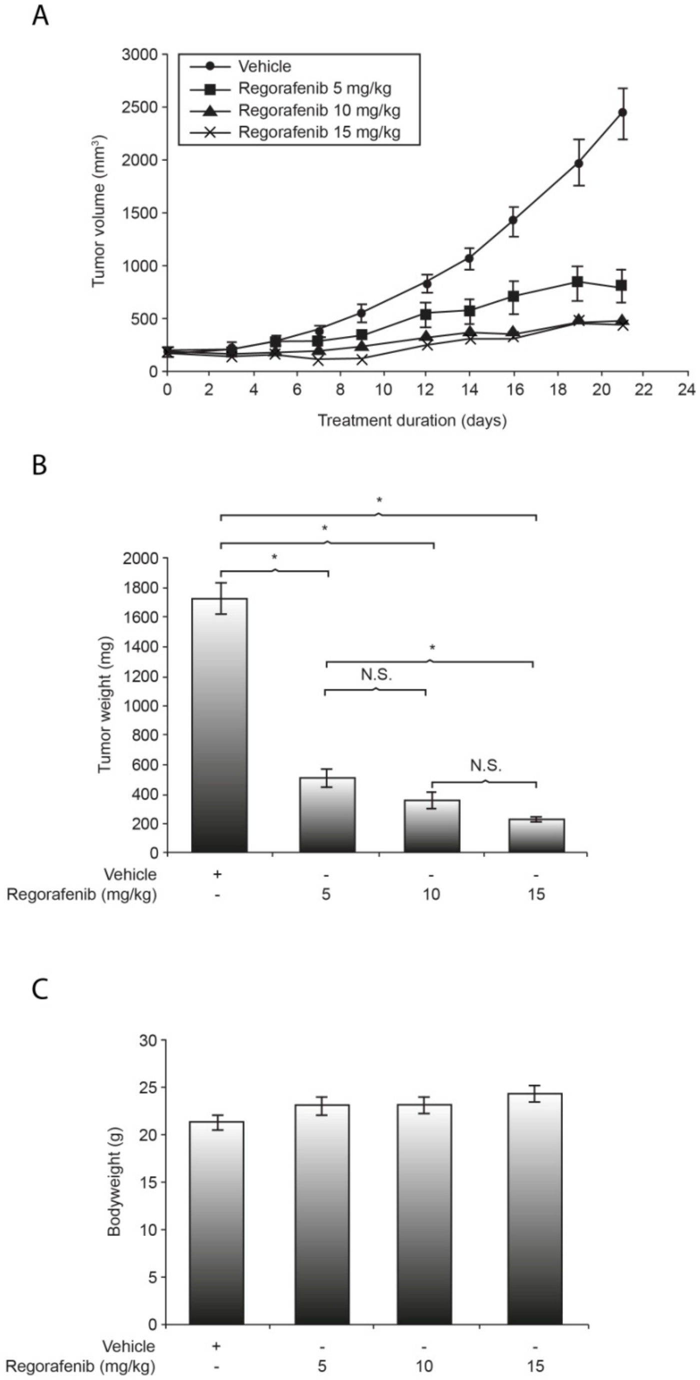

Supplement: Additional file 2: Figure S1. — Dose-dependent effects of regorafenib 5, 10, and 15 mg/kg/day on tumor growth inhibition (A), tumor weight (B), and bodyweight (C) in xenograft model GC28-1107. (PDF 143 kb) [file 13046_2015_243_MOESM2_ESM.pdf]
