# Supplementary material for: Antitumor activity of the multikinase inhibitor regorafenib in patient-derived xenograft models of gastric cancer
Source: J Exp Clin Cancer Res. 2015 Oct 29;34:132. doi: 10.1186/s13046-015-0243-5 (PMC4625870; doi:10.1186/s13046-015-0243-5)

Supplementary Figure 2. Effects of regorafenib 10 mg/kg/day on tumor growth in xenografts GC22-0808, GC23-0909, GC30-0309, GC-10-0608, and GC05-0208B. Data shown are mean ± standard error.

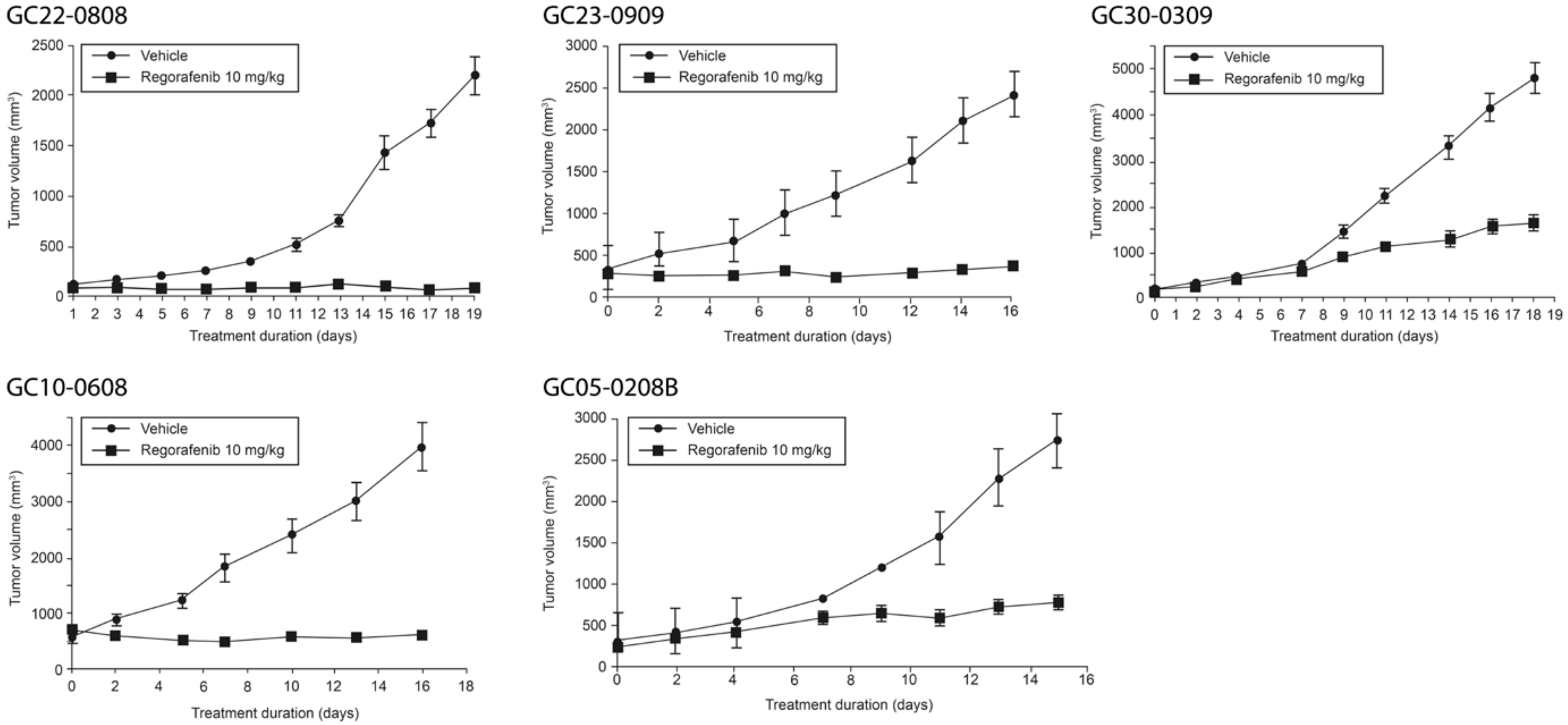

Supplement: Additional file 3: Figure S2. — Effects of regorafenib 10 mg/kg/day on tumor growth in xenografts GC22-0808, GC23-0909, GC30-0309, GC-10-0608, and GC05-0208B. (PDF 75 kb) [file 13046_2015_243_MOESM3_ESM.pdf]
