# Supplementary material for: Antitumor activity of the multikinase inhibitor regorafenib in patient-derived xenograft models of gastric cancer
Source: J Exp Clin Cancer Res. 2015 Oct 29;34:132. doi: 10.1186/s13046-015-0243-5 (PMC4625870; doi:10.1186/s13046-015-0243-5)

Huynh H, Ong R, Zopf D. Antitumor Activity of the Multikinase Inhibitor Regorafenib in Patient-Derived Xenograft Models of Gastric Cancer

Supplementary Figure 3. Effects of regorafenib on TIE2 expression in gastric cancer xenograft model GC05-0208B.

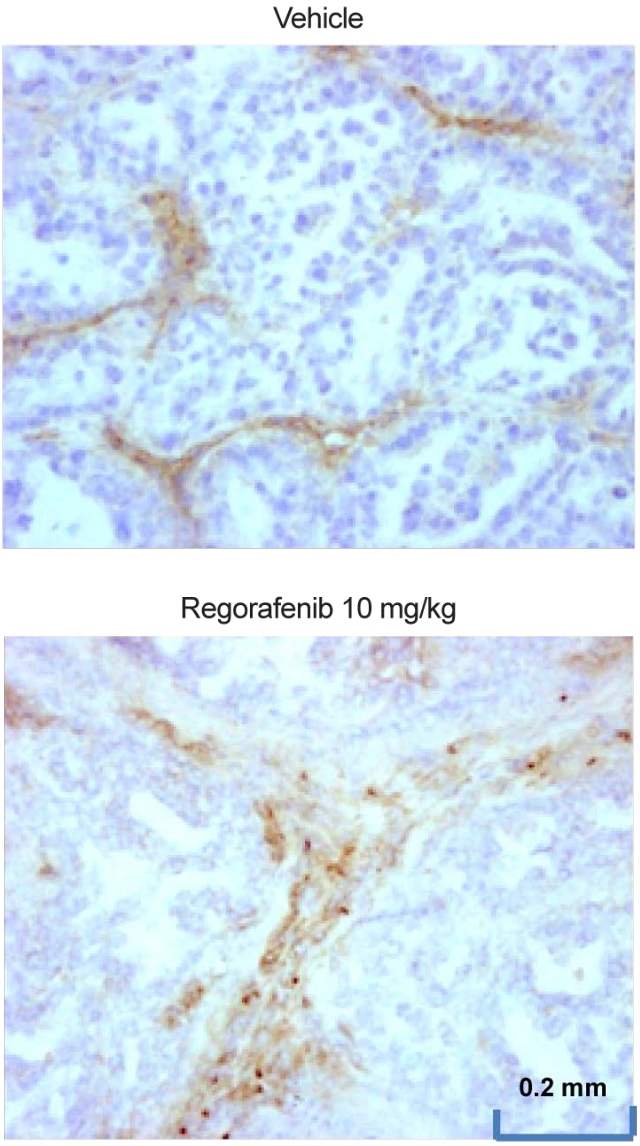

Supplement: Additional file 4: Figure S3. — Effects of regorafenib on TIE2 expression in gastric cancer xenograft model GC05-0208B. (PDF 94 kb) [file 13046_2015_243_MOESM4_ESM.pdf]
